# The impact of economic growth target management on urban green land utilization efficiency

**DOI:** 10.1371/journal.pone.0321779

**Published:** 2025-04-18

**Authors:** Yu Wang

**Affiliations:** Harbin University of Commerce, Harbin, Heilongjiang, PR China; Huazhong University of Science and Technology, CHINA

## Abstract

Improving urban green land utilization efficiency (UGLUE) is the key to promoting green and sustainable development in China. Clarifying the impact of economic growth target management (EGTM) on UGLUE and its mechanism of action is of great significance to improving UGLUE. Selecting 273 cities in China from 2010 to 2021 as the research sample, this paper uses panel data model, and spatial Durbin model (SDM) to empirically examine the impact, transmission mechanism and spatial spillover effect of EGTM (including economic growth target values, hard constraints and soft constraints of economic growth targets) on UGLUE. In addition, this paper uses panel threshold model to verify the threshold role of environmental regulation in the relationship between EGTM and UGLUE. The research found that: (1) Local economic growth target value and its hard constraints have a negative impact on UGLUE, while soft constraints are conducive to improving UGLUE. (2) Green technology innovation and industrial structure upgrading are the main transmission channels. (3) As the intensity of environmental regulation increases, the negative impact of economic growth target value and its hard constraints on UGLUE weakens, while the positive impact of its soft constraints on UGLUE strengthens. (4) The economic growth target value and its hard constraints of surrounding areas can reduce the UGLUE in the region, while its soft constraints can improve the UGLUE in the region. (5) Economic growth targets have the greatest negative impact on UGLUE in the central region and resource-based cities. In the future, the importance of GDP growth rate in official performance evaluations should be reduced. More flexible “soft constraints” should be used to set economic growth targets.

## 1. Introduction

With the continuous advancement of industrialization and urbanization, the area of urban construction land in China has expanded rapidly [1]. Statistical data show that from 2006 to 2020, the area of urban construction land in China has increased from 31765.70 km^2^ to 58355.3 km^2^, with an average annual growth rate of 4.44% [2]. The rapid expansion of urban construction land has led to issues such as economic stagnation, a shortage of arable land, and environmental pollution, which contradict the strategy of promoting sustainable development [3]. In order to control the expansion speed of urban construction land, China has introduced a strict land use system, requiring local governments not to exceed the scale of urban construction land without authorization. How to increase economic output and promote sustainable development in China with limited land resources is an urgent problem to be solved in various regions. UGLUE refers to the maximization of economic, social and ecological benefits with the lowest input of land factors [4]. It embodies the core concept of sustainable development [5], that is, the sustainable unity of economy, society and environment. Only by improving UGLUE and promoting the sustainable use of land, can sustainable development be truly realized.

In the past, scholars have explored ways to improve UGLUE from the perspectives of economic factors [6], social factors [7], industrial factors [8], and technological factors [9]. In recent years, scholars have shifted their research perspective to policy factors at the macroeconomic level and began to pay attention to their impact on UGLUE [10]. However, no research has yet incorporated the policy factor of EGTM into the analytical framework of UGLUE. EGTM refers to the higher-level government incentivizing winning the promotion tournament by setting relevant targets [11]. To win the “promotion tournament”, local government officials set more aggressive local economic growth targets based on the economic growth targets set by central government. To achieve this goal, local governments allocate resources to areas that can quickly achieve economic growth goals [12]. This leads to a large number of urban land being used inefficiently, thus affecting UGLUE. Based on this, what is the impact of EGTM on UGLUE? What is its mechanism of action? Answering these questions not only provides new clues for understanding the extensive use of urban land caused by the acceleration of economic growth, but also provides policy implications for improving the government’s EGTM system.

Based on this, this paper innovatively incorporates EGTM and UGLUE into the same analytical framework. Based on theoretical analysis, this paper uses panel data of 273 cities in China from 2010 to 2021, panel regression model, mediation effect model and SDM to empirically analyze the direct impact, mediation effect and spatial spillover effect of EGTM on UGLUE. Using the panel threshold model to test the threshold effect of environmental regulation in the relationship between the two. The results show that: (1) The improvement of local economic growth goals and the adoption of hard constraints have a negative impact on UGLUE, while the soft constraints are conducive to improving UGLUE. Its transmission channels are green technology innovation and industrial structure upgrading. (2) With the increase of environmental regulation intensity, the negative impact of economic growth target and its hard constraints on UGLUE is weakened. The positive impact of soft constraints on UGLUE is enhanced. (3) EGTM has a positive spatial spillover effect.

The marginal contribution of this paper lies in: First, in terms of research perspective, previous studies have studied the influencing factors of UGLUE from the perspectives of economic agglomeration, facility construction, technological innovation, lacking attention to the effect of EGTM. From the perspective of EGTM, this paper examines the impact of EGTM on UGLUE and its mechanism of action for the first time, broadening the research boundary of land use. Second, in terms of research methods, the existing studies on EGTM use ordinary panel regression models that do not consider spatial spillover effects for empirical analysis. However, in fact, the local government’s economic growth target setting has the benchmarking competition characteristics of mutual reference imitation between cities. This paper introduces the SDM to explore the spatial spillover effect of EGTM on UGLUE, which helps to better understand the interaction strategy of target management between regions. Third, in terms of research samples, most of the existing studies on EGTM remain at a single level such as national, inter-provincial, typical cities and specific industries. There are few studies on the overall cities. Considering the fierce competition between different prefecture-level cities in the same province, this paper takes EGTM specific to the city level. Taking 273 cities in China as research samples to examine the impact of local government behavior on UGLUE in more detail.

## 2. Literature review

UGLUE refers to maximizing land economic output at the cost of minimizing ecological and environmental losses under the constraints of urban land resources[13]. Related research mainly includes two aspects: The measurement and influencing factors of UGLUE. First, the measurement of UGLUE. The measurement subjects mainly include provinces [14], cities [15] and extensions. The measurement method has transitioned from a single indicator measurement method [16] to a multi-indicator comprehensive measurement method [17]. Since the measurement of UGLUE is not limited to economic results, but also covers social, ecological and other indicators, scholars use a multi-indicator comprehensive measurement method to measure UGLUE. Some scholars used stochastic frontier analysis (SFA) to measure UGLUE [14]. However, since SFA requires a specific form and the assumptions are too strict, it has certain limitations in measuring UGLUE. Therefore, some scholars began to use data envelopment analysis (DEA) to measure UGLUE [18–22]. Second, the factors affecting UGLUE. The factors affecting UGLUE can be divided into two categories: Internal resources and external environment. Internal resources mainly include transportation infrastructure [23], industrial structure [24], urban structure [25], intelligence level [26], etc. These factors come from the inherent development conditions of city. The external environment mainly includes land transfer [27], land finance [28], policy pilot [29], and environmental regulation [30, 31]. These factors come from the government’s institutional arrangements. However, no research has focused on the relationship between the institutional arrangement factor of EGTM and UGLUE.

EGTM serves as a crucial instrument for fostering swift economic expansion across various nations. At present, research on the policy effects of EGTM mainly focuses on its impact on economic growth [32], government spending [33], environmental pollution [34], energy consumption [35], and technological innovation [36]. No research has shifted its perspective to land and explored the impact of economic growth targets on UGLUE. In terms of research methods, scholars mainly use the least squares method (OLS) [37], the iterative three-stage least squares method (3SLS) [38], and the fixed effect model [39] to empirically analyze the policy effectiveness of economic growth targets. The horizontal competition phenomenon of local governments in setting economic growth targets is not fully considered. In terms of sample selection, the research on economic growth targets mainly stays at the single level of countries [33], provinces [32], and specific industries [40]. There are few studies on the city as a whole, which ignores the fierce competition between different prefecture-level cities and the heterogeneous role of economic growth targets in the process of changing land use patterns.

## 3. Theoretical analysis and research hypotheses

### 3.1. The direct impact of local EGTM on UGLUE

**Fig 1:**
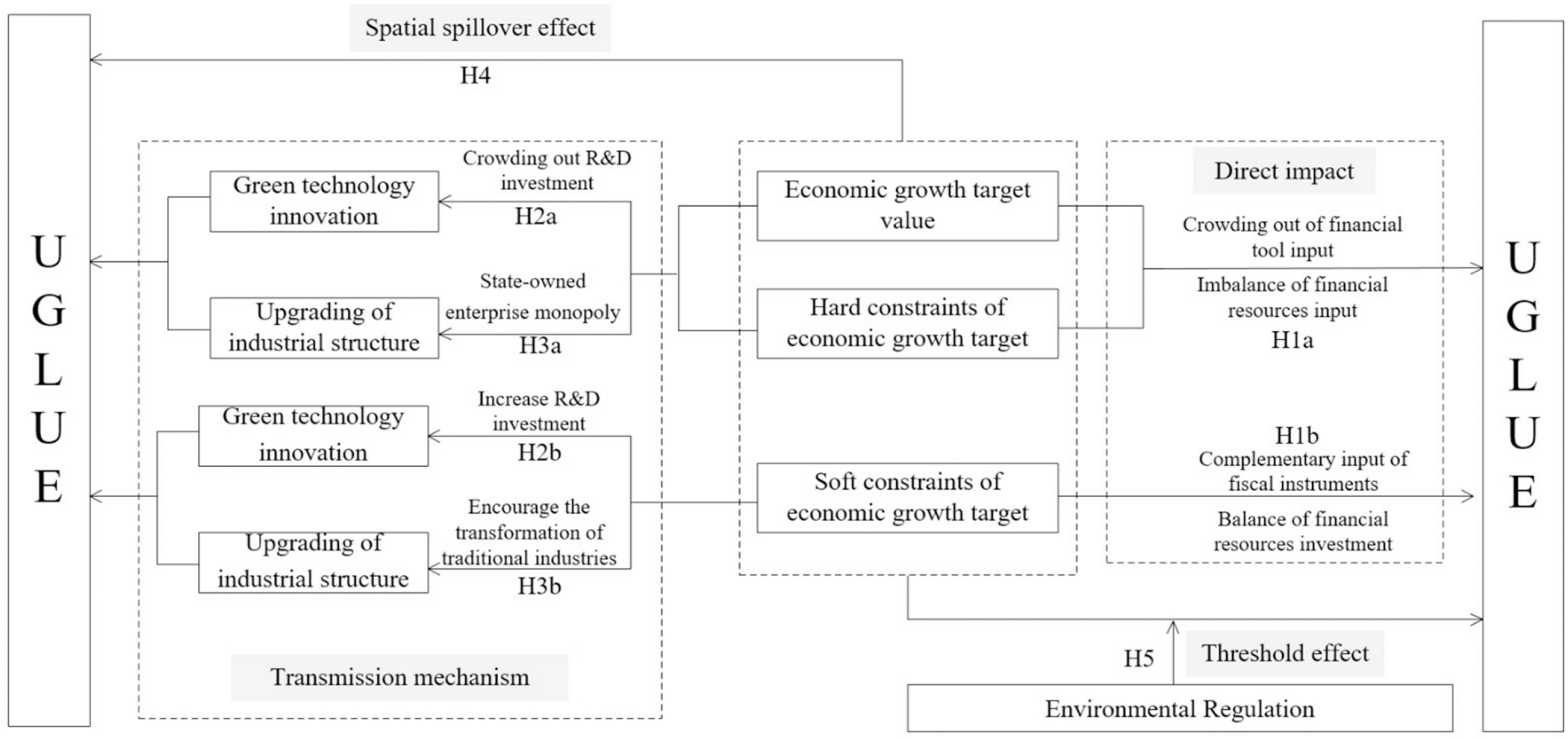
The framework of theoretical analysis.

Public choice theory suggests that government officials are ‘rational economic men’ who not only aim to maximize social welfare when making policy decisions but also pursue their own interests [41]. Under China’s’ promotion tournament’ model, which uses GDP as an assessment indicator, the EGTM system serves as a significant tool for local governments to enhance economic growth within their respective areas [42]. In order to maximize their own political interests during their term, local government officials often set economic growth targets that deviate from actual conditions, thereby hindering the improvement of UGLUE (Fig 1).

The impact of local EGTM on UGLUE is specifically reflected in the following two aspects: First, the crowding-out effect of policy input tools. In the context of fiscal decentralization, local governments need to achieve their economic growth targets through various policy tools (such as tax tools, expenditure tools, and environmental governance tools). Although these tools can help local governments achieve their economic growth targets in the short term, they also lead to an imbalance in the allocation of local fiscal resources, which is not conducive to improving UGLUE. Regarding local fiscal revenue and environmental governance expenditure, when local governments take measures such as reducing tax rates or providing tax incentives to stimulate economic growth, their fiscal revenue may decrease in the short term. The limited fiscal resources lead to cuts in investment in green infrastructure and environmental protection projects. Concerning the imbalance in resource allocation, when local governments set excessively high economic growth targets, they tend to prioritize the use of land for industrial development and infrastructure projects over ecological initiatives like parks and green spaces [43]. This biased land use can reduce environmental quality. Second, the structural effect of fiscal resource investment. Local governments tilt resources to support productive projects with short cycles and quick results, while ignoring innovative investments with long cycles and slow results. An investment structure that “emphasizes production and neglects innovation” has been formed, which aggravates environmental pressure. In addition, as a large amount of financial resources flow into traditional industries, fiscal expenditures for environmental protection are squeezed out. Effective control of environmental pollution and ecological restoration work are difficult to carry out, thereby reducing UGLUE.

In their annual government work reports, local governments typically establish specific economic growth targets, often accompanied by adverbs indicating varying levels of intensity. For example, expressions such as “strive to” and “ensure” reflect hard constraints, indicating that local governments are more committed to achieving these targets. Local governments take short-term economic measures to quickly achieve economic growth targets, which reduces UGLUE by squeezing out funds for environmental protection projects. The use of “left and right” and “up and down” reflects the soft constraints characteristics, providing more flexibility for the realization of economic growth targets. While pursuing economic growth, local governments can also consider improving environmental quality [44], thereby improving UGLUE. Specifically, local governments do not have to rely too much on short-term economic measures to achieve their goals quickly. On the contrary, local governments are able to invest part of their resources in green infrastructure and environmental projects that are critical to improving UGLUE [45]. In addition, local governments have more autonomy to optimize the allocation of financial resources. By increasing investment in innovative and livelihood projects and optimizing the investment structure, they can enhance the ecological value of land resources. The following hypothesis is proposed:

**Hypothesis 1a** Local economic growth target value and its hard constraints have a negative impact on UGLUE.

**Hypothesis 1b** The soft constraints of “leaving room” for economic growth targets have a positive impact on UGLUE.

### 3.2. Transmission mechanism

#### 3.2.1. Green technology innovation.

The setting of economic growth target value and its hard constraints have a “crowding-out effect” on the R&D investment in green technologies, thereby inhibiting green technology innovation. From the perspective of corporate financing, under the pressure of economic growth targets, the government allocates a substantial quantity of economic resources to infrastructure construction [35]. As the available funds in the market decrease, financial institutions raise interest rates to balance the supply and demand for funds. The financing costs of enterprises increase. Since R&D activities are characterized by high investment, long cycle and high risk, they put tremendous pressure on corporate cash flow [46]. Compared with long-term R&D investment, companies may be more inclined to invest in short-term projects. In addition, firms with high debt are more likely to cut R&D spending when funding is tight to reduce risks [47]. From the perspective of private investment, local governments provide robust support for the real estate industry, leading to an increase in housing prices. Driven by high profits, companies have a strong arbitrage motivation. They withdraw their own capital from green technology development and invest it in the real estate market [48]. Innovation funds are squeezed, hindering green tech innovation.

In the context of China’s economic transformation, the central government advocates slowing down the pace of economic growth and focusing on the quality of economic growth. Some regions begin to set soft constraints on economic growth targets. Soft constraints make the government no longer blindly pursue economic growth and scale, but take into account the protection of environment. Green innovation is important for environmental protection [49]. Local governments invest financial resources in the field of green innovation through financial subsidies, tax incentives, technical support and other measures. Enterprises are encouraged to carry out green technology innovation activities. This incentive effect can promote cooperation between enterprises. Through resource sharing, experience exchange and collaborative R&D, enterprises can achieve complementary advantages and jointly cope with the challenges in the process of green technology innovation.

Technological innovation theory holds that innovation is an important driving force for endogenous economic growth [50]. Green technology innovation can reduce fossil energy consumption and increase the use of clean energy in the front-end production process [51]. Improve the degree of pollutant purification from the end-of-pipe treatment and reduce pollutant emissions. In the process of land use, both economic and environmental benefits can achieve synergistic growth, thereby improving UGLUE. The following hypothesis is proposed:

**Hypothesis 2a** Local economic growth target value and its hard constraints inhibit green technology innovation, which in turn has a negative impact on UGLUE.

**Hypothesis 2b** Soft constraints can improve UGLUE by promoting green technology innovation.

#### 3.2.2. Upgrading of industrial structure.

Economic growth targets value and its hard constraints hinder industrial upgrading via state-owned monopolies and infrastructure. Regarding the monopoly of the state-owned economy, local governments excessively allocate production factors such as capital and land toward state-owned enterprises that lack the motivation for innovation [52]. Emerging and green industries cannot develop due to lack of financial and policy support. The pace of upgrading the entire industrial structure slows down. Regarding infrastructure construction, the government invests funds in “short-cycle, low-risk” infrastructure construction, driving the expansion of manufacturing industries such as steel and cement that already have overcapacity problems. The upgrading of traditional extensive industries is inhibited.

When the economic growth target setting changes from “hard constraints” to “soft constraints”, the government considers environmental protection. The economic growth mode changes from factor-driven to innovation-driven, which is conducive to industrial upgrading. Specifically, the government encourages enterprises to carry out green production through a series of incentives, such as tax incentives, financial support and technological innovation incentives. The traditional industry is gradually transforming to the intelligent and green direction. Meanwhile, the government also strengthens the supervision of high pollution and high emission industries. By raising environmental standards and strengthening law enforcement, these industries are forced to carry out technological transformation or gradually withdraw from the market. Environmental protection industry has a huge space for development.

The upgrading of industrial structure has a “structural dividend” effect [53]. It can promote the concentration of low-pollution industries in the city center and the relocation of traditional high-pollution industries to the peripher. The economic and environmental benefits of urban land achieve coordinated development. The following hypothesis is proposed:

**Hypothesis 3a** Local economic growth target values and its hard constraints inhibit the upgrading of industrial structure, which has a negative impact on UGLUE.

**Hypothesis 3b** Soft constraints can improve UGLUE by promoting the upgrading of industrial structure.

### 3.3. Threshold effect

Environmental regulation plays a key role in the impact of local EGTM on UGLUE. When the intensity of environmental regulation is low, local governments prioritize economic growth and focus on the economic benefits of land while ignoring their environmental benefits. In allocating resources, local governments invest more, local governments invest more resources in high-pollution areas that can rapidly promote economic growth and reduce UGLUE. As the intensity of environmental regulation increases, the government’s short-term economic behavior is constrained by environmental regulation. The negative impact of economic growth targets on UGLUE is weakened. From the government’s perspective, when the intensity of environmental regulation is high, the government sets up environmental barriers for enterprises when attracting investment. Highly polluting and high-emission industrial enterprises are prohibited from entering [54]. In addition, local governments also implement tax incentives and financial subsidies for low-pollution, high-output enterprises. These preferential policies can motivate enterprises to boost their investment in green production. From the perspective of enterprises, stronger environmental regulations produce the “Porter effect” [55], prompting enterprises to innovate clean technologies. The green land use pattern is formed. Hypothesis 4 is proposed:

**Hypothesis 4** The impact of local EGTM on UGLUE is affected by the threshold effect of environmental regulation intensity. As the intensity of environmental regulation increases, the inhibitory effect of local economic growth targets value and their hard constraints on UGLUE weakens. The promotion effect of soft constraints on UGLUE increases.

### 3.4. Spatial spillover effect

Higher-level governments struggle to set clear, objective criteria for evaluating local governments. As a result, local governments are allowed to compete independently and the winners are selected by comparing the competition results. A top-down benchmarking competition assessment model is formed [56]. In this model, local governments respond strategically to the economic growth targets of governments at the same level. A race for economic growth targets is formed among local governments [57].

Faced with tremendous pressure for economic growth, local governments are forced to adopt a series of radical policies to stimulate economic growth. They invest financial resources in infrastructure construction and traditional industrial projects, marginalizing ecological projects such as green infrastructure and parks. In addition, the misallocation of financial resources also makes it difficult to carry out ecological restoration work, thereby reducing the UGLUE of local region. Hypothesis 5 is proposed:

**Hypothesis 5** Local EGTM has a spatial spillover effect on UGLUE. The increase in economic growth targets value and hard constraints in surrounding areas reduce the UGLUE of local region. The soft constraints on economic growth targets in surrounding areas increase the UGLUE of local region.

## 4. Research methods and variable selection

### 4.1. Research methods

#### 4.1.1. Panel regression model.

The following model is constructed in this study:


UGLUEit=α0+β0EGTMit+ηXit+λi+εit (1)

Among them, *UGLUE*_*it*_ represents the green utilization efficiency of urban land in city *i* in year *t*. *EGTM*_*it*_ represents the economic growth target management of city *i* in year *t*, including economic growth target value, hard constraints and soft constraints. *X*_*it*_ represents the control variable. λi is the individual effect. εit is the residual term. Since the economic growth target value (EG), hard constraints (EHC) and soft constraints (ESC) are all macro time series variables. If the year fixed effect is controlled in the model, the impact of EGTM on UGLUE may be absorbed by year fixed effect. Therefore, referring to the prior literature [58], this paper adopts the regional fixed effect model instead of year-region two-way fixed effect model. Considering that not controlling the year fixed effect may omit some variables that change over time, this paper adds macroeconomic variables to the model. These include economic development level, degree of openness to the outside world, and population size, which alleviate the problem of omitted variables that may exist in the time section.

#### 4.1.2. Panel threshold model.

Considering the possible threshold effect of economic development goals on UGLUE, this paper constructs the following model:


UGLUEit=α3+β3EGTMitIit(ER≤γ1)+β4EGTMitIit(ER>γ1)+ηXit+λi+εit (2)

Among them, *I()* is the indicative function. *ER* is the threshold variable of environmental regulation. γ1 is the specific threshold value to be estimated.

#### 4.1.3. Spatial Durbin model.

This paper uses SDM to examine the possible spatial spillover effects of EGTM on UGLUE. The model is set as follows:


$$UGLUEit=\alpha 4+\chi 0\sum_{j}WijUGLUEit+\beta 5EGTMit+\beta 6\sum_{j}WijEGTMit++\eta Xit+\lambda i+\varepsilon it$$ (3)

*W*_*ij*_ is the spatial weight matrix. The geographical distance weight matrix is regarded as spatial weight matrix. χ0 is the spatial autocorrelation coefficient. β6 is the estimated coefficient of the impact of the EGTM of surrounding area on UGLUE in this area.

### 4.2. Variables selection

#### 4.2.1. Explained variables.

UGLUE: This paper uses the super-efficiency SBM model considering non-expected output to measure UGLUE. The specific formula is as follows:


minρ=1−1n∑i=1nsi−xik1−1θ1+θ2(∑r=1θ1srgyrkg+∑r=1θ2srbyrkb)

$$\begin{array}{l} s.t.X\lambda +s-=xk\\ Yg\lambda -sg=ykg\\ Yb\lambda -sb=ykb\\ \lambda ,s-,sg,sb\geq \text{0} \end{array}$$ (4)

Among them, s=(s−,sb,s+) is the slack of input, expected output and undesired output; *ρ* is the efficiency value of the decision-making unit (DMU), which ranges from 0 to 1. When ρ=1, that is, when s−=sb=s+=0, the DMU is effective, otherwise the DMU is invalid.

Based on the super-efficiency SBM, this paper constructs the following global Malmquist index:


$$GMLt,t+1(xt,yt,bt,xt+1,yt+1,bt+1)=\left\{\begin{array}{l} \frac{1+\vec{DCT}(xt,yt,bt;gt)}{1+\vec{DCT}(xt+1,yt+1,bt+1;gt+1)}\\ \times \frac{1+\vec{DCT+1}(xt,yt,bt;gt)}{1+\vec{DCT+1}(xt+1,yt+1,bt+1;gt+1)} \end{array}\right\}\frac{1}{2}$$ (5)

Among them, GMLt,t+1 is the global Malmquist index from period *t* to period *t+1*. GMLt,t+1>1 indicates that compared with period *t*, the UGLUE in period *t+1* is improved. GMLt,t+1<1 indicates that compared with period *t*, the UGLUE in period *t+1* is reduced.

Table 1 shows the input–output indicators.

**Table 1:** Input–output indicators.

Type	Name	Description	Mean	Std. Dev.	Min	Max
Input	Land	Construction land area (km^2^)	149.01	213.42	1	2364.50
	Capital	Fixed capital stock (100 million yuan)	104000000	112000000	426600	1140000000
	Labor	Number of employees in the secondary and tertiary industries (10,000 people)	407738.50	957391.40	15600	16000000
Expected output	Economic benefits	Value added of secondary and tertiary industries (yuan)	1337.03	1993.11	0.79	19162.56
	Social benefits	Average wage of urban employees (yuan)	49700.87	18797.98	3959.38	487091.60
	Ecological benefits	Green coverage rate of built-up areas (%)	39.92	6.68	0.36	100
Unexpected output	Pollutant emissions	Industrial wastewater discharge (10,000 tons)	6121.69	8133.78	7	101439.50
		Industrial sulfur dioxide emissions (tons)	38122.75	47567.26	0	586117
		Industrial smoke (dust) emissions (tons)	28401.15	126131.30	32	5168812

#### 4.2.2. Explanatory variables.

EGTM: This paper examines EGTM from two aspects: Economic growth target value and its constraints. The economic growth target value is expressed as the target value mentioned in the work report of each city government [38]. Economic growth target constraints include hard constraints and soft constraints. This paper uses the modal particles used in the city government reports when announcing growth targets as identification variables [37]. Economic growth targets that use words such as “above”, “exceed”, “higher than”, and “ensure” are considered hard constraints, while economic growth targets that use modal particles such as “around”, “up and down”, and “between” are considered soft constraints.

#### 4.2.3. Threshold variables.

Environmental regulation: There are two measurement methods. One is to characterize it through the frequency of environmental protection vocabulary and pollution control investment mentioned in local documents [59]. The other is to reversely infer environmental regulation through pollution control results and reflect the level of regional environmental regulation by pollutant emissions [60]. This paper uses the frequency of environmental protection words in city government work reports obtained through text analysis to measure environmental regulation. Environmental protection vocabulary includes environmental protection, environmental protection, pollution, energy consumption, emission reduction, pollution discharge, ecology, green, low carbon, air, chemical oxygen demand, sulfur dioxide, carbon dioxide, PM10 and PM2.5, etc.

#### 4.2.4. Control variables.

(1) Economic development level. On the one hand, local governments, in pursuit of short-term interests, vigorously develop highly polluting industries that rely on resource development. The development of these industries consumes a lot of land and produces pollutants, reducing UGLUE. On the other hand, with the improvement of economic development level, the ability of local governments and enterprises to invest in land sustainable development technologies increases. Land use patterns have shifted from extensive to intensive [61]. This paper uses GDP per capita as a measurement indicator.
(2) Degree of openness. Opening up to the outside world enables advanced land management and environmental protection technologies to spread across regions, resulting in technological spillover effects. Through the spillover effect, various regions absorb these technologies and transform them into environmentally friendly technologies suitable for their own development, promoting sustainable land development. However, according to the pollution paradise hypothesis, increased openness to the outside world may also increase the risks of extensive land use and environmental pollution [6]. This article uses the ratio of total imports and exports to GDP to express it.
(3) Degree of labor distortion. The distortion of labor market makes it impossible for labor market to provide labor with environmental protection and land management skills. Urban land cannot be used effectively. In addition, the concentration of labor resources in pollution-intensive industrial sectors can increase pollutant emissions and reduce UGLUE. Referring to the research of Chen et al. [62], this paper measures the degree of labor distortion (DL_it_).

$$DLit=\frac{1}{1+\gamma Li}$$

$$\gamma Li=\left(\frac{Li}{L}\right)/\left(\frac{Si\beta Li}{\beta L}\right)$$

$$Si=PiYi/Y,\;\beta L=\sum_{i}^{N}Si\beta Li$$ (8)

Among them, *S*_*i*_ represents the proportion of the output of city *i* in the total output *Y*. $\frac{Li}{L}$ represents the proportion of the labor force used by city *i* in the total labor force. $\frac{Si\beta Li}{\beta L}$ is the proportion of labor used by city *i* when the labor force is effectively allocated. βLi is the labor output elasticity of each city estimated using the production function. To ensure the consistency of the estimation results, we take the absolute value of degree of labor distortion.

(4) Population size. On the one hand, the expansion of population size may produce “scale effect” and “agglomeration effect” [63], improving UGLUE. On the other hand, an increase in population size also leads to overexploitation of land resources and damage to the ecological environment [7], reducing UGLUE. This paper uses the total population as a measure of population size.

### 4.3. Data sources and descriptive statistics

Taking 273 cities in China from 2010 to 2021 as a sample, this study explores the impact of EGTM on UGLUE. Among them, data on economic growth targets and environmental regulations come from the work reports of prefecture-level city governments. Green patent data come from the patent search platform of the State Intellectual Property Office. Other data come from the China Statistical Yearbook, China City Statistical Yearbook, and China Urban Construction Statistical Yearbook. Interpolation and linear fitting methods are used to fill in missing values in some prefecture-level city data. The monetary price index is deflated based on the price index with 2010 as the base year. To eliminate the impact of heteroskedasticity, I logarithmize GTI, GDPP, and POP. Table 2 shows the descriptive statistics of variables.

**Table 2:** Descriptive statistics of variables.

	Variables	Meaning	Obs	Mean	Std.Dev	Min	Max
Explained variables	UGLUE	Urban green land use efficiency	3,276	1.1076	0.5041	0.2697	3.2344
Explanatory variables	EG	Economic growth target value	3,276	9.5383	3.0496	1.0000	25.0000
	EHG	Hard constraints on economic growth targets	3,276	0.7647	0.4243	0.0000	1.0000
	ESC	Soft constraints on economic growth targets	3,276	0.3758	0.4844	0.0000	1.0000
Threshold variables	ER	Environmental Regulation	3,276	0.0034	0.0014	0.0003	0.0124
Control variables	GDPP	Economic development level (logarithm)	3,276	10.1246	0.5533	8.4647	12.6831
	OPEN	Degree of openness	3,276	0.1737	0.2781	0.0000	2.4498
	LD	Degree of labor distortion	3,276	0.7679	0.9834	0.0001	36.2601
	POP	Population size (logarithm)	3,276	5.8987	0.7024	2.9704	8.1362

## 5. Results and discussion

### 5.1. Spatial-temporal characteristics of UGLUE

#### 5.1.1. Time trend.

**Fig 2:**
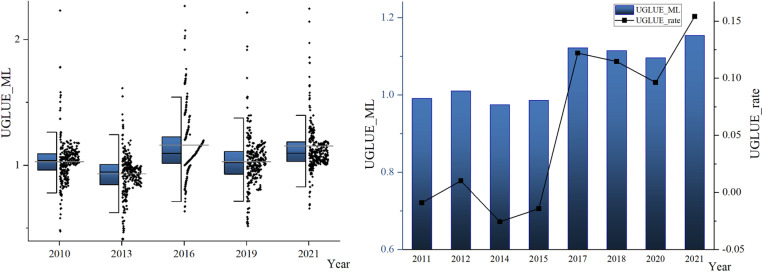
ML mean of UGLUE in China from 2010 to 2021.

Fig 2 shows the changing trend of UGLUE based on the ML index from 2010 to 2021. The ML mean is 1.0498, indicating that from 2010 to 2021, UGLUE has shown an overall upward trend. This trend is due to the increasing demand for land resources caused by the accelerated urbanization process. In the case of limited land resources, policymakers have to focus on optimizing land management and promoting sustainable land use. In addition, the public pays more and more attention to the quality of the living environment, which prompts them to pay more attention to how to improve environmental quality through green land use. The UGLUE declined in 2010–2011 and 2012–2015. During this period, China’s economy may still be in a slow recovery stage from financial crisis. Under economic pressure, regions may pay more attention to short-term economic growth at the expense of environmental protection. The enforcement of land use-related policies has weakened.

#### 5.1.2. Spatial characteristics.

**Fig 3:**
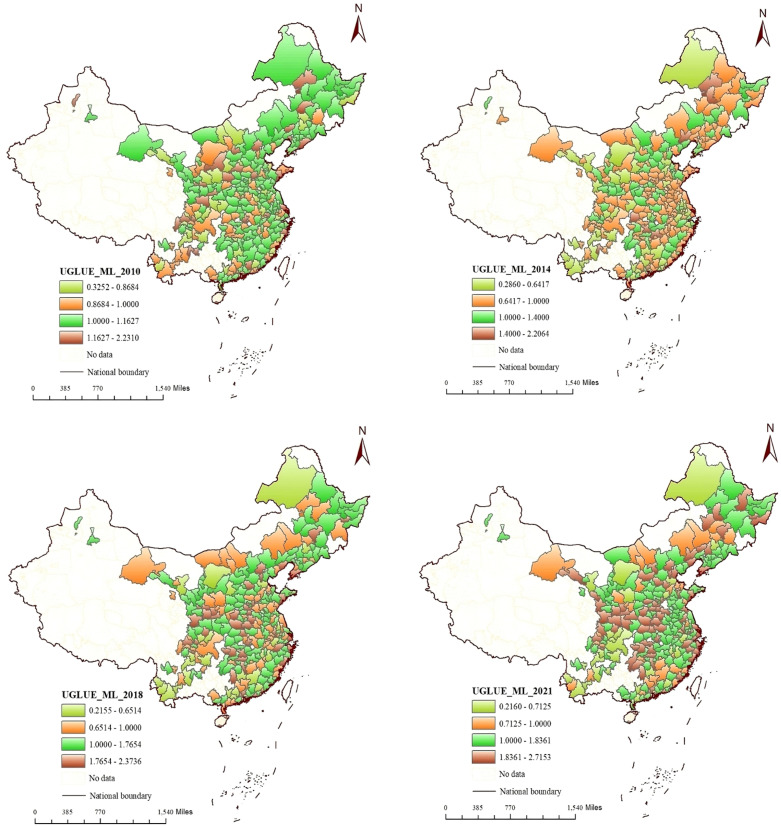
Spatial distribution of UGLUE in China in 2010, 2014, 2018 and 2021 (Map created using ArcGIS 10.2, http://www.esri.com/software/arcgis **).**

Fig 3 shows the spatial distribution of UGLUE in China in 2010, 2014, 2018 and 2021. More and more cities have achieved growth in UGLUE. In 2021, the number of cities with increased UGLUE reaches 231. Most urban land has been effectively utilized. However, the UGLUE in some cities such as Chongqing, Shenzhen and Nanjing is still on a downward trend. The land use mode needs to be changed urgently.

### 5.2. Benchmark regression results

The benchmark regression results are shown in Table 3. Based on test results, we choose the estimation results of fixed effect model for analysis. The estimated coefficients of economic growth target value and its hard constraints are -0.0346 and -0.0309 respectively, which indicates that both reduce UGLUE.

**Table 3:** Benchmark regression results.

	(1)OLS	(2)FE	(3)RE	(4)OLS	(5)FE	(6)RE	(7)OLS	(8)FE	(9)RE
EG	-0.0347***	-0.0346***	-0.0331***						
	(0.0027)	(0.0023)	(0.0022)						
EHC				-0.0250	-0.0309*	-0.0337*			
				(0.0205)	(0.0174)	(0.0172)			
ESC							0.0224	0.0302**	0.0331**
							(0.0180)	(0.0153)	(0.0151)
lnGDPP	0.152***	0.375***	0.233***	0.148***	0.165***	0.169***	0.147***	0.169***	0.169***
	(0.0182)	(0.0498)	(0.0315)	(0.0187)	(0.0493)	(0.0319)	(0.0187)	(0.0494)	(0.0319)
OPEN	-0.119***	-0.0586	-0.179***	-0.106***	-0.230***	-0.213***	-0.106***	-0.223***	-0.211***
	(0.0355)	(0.0787)	(0.0564)	(0.0363)	(0.0805)	(0.0577)	(0.0363)	(0.0806)	(0.0577)
LD	0.0075	-0.0161**	-0.0105	0.0162*	-0.0067	-0.0015	0.0162*	-0.0070	-0.0017
	(0.0088)	(0.0080)	(0.0079)	(0.0089)	(0.0083)	(0.0081)	(0.0089)	(0.0083)	(0.0081)
lnPOP	0.0944***	0.730***	0.125***	0.1000***	0.741***	0.130***	0.100***	0.741***	0.130***
	(0.0121)	(0.125)	(0.0277)	(0.0124)	(0.130)	(0.0282)	(0.0124)	(0.130)	(0.0282)
Constant	-0.645***	-6.646***	-1.635***	-0.953***	-4.867***	-1.306***	-0.968***	-4.946***	-1.342***
	(0.203)	(0.935)	(0.365)	(0.208)	(0.959)	(0.372)	(0.207)	(0.960)	(0.372)
F test		54.98***			12.67***			12.81***	
Hausman test		72.86***			32.56***			32.54***	
LM test			2940.69***			2787.30***			2789.70***
R^2^	0.082	0.048		0.039	0.021		0.039	0.021	
N	3276	3276	3276	3276	3276	3276	3276	3276	3276

Standard errors in parentheses.

***p < 0.01.

**p < 0.05.

*p < 0.1.

This conclusion is consistent with existing research. In order to achieve economic growth targets, local governments tend to invest in infrastructure projects with short cycles and quick results [64], which creates huge demand for highly polluting raw materials such as building materials, steel, and cement, exacerbating environmental pollution. In addition, government investment in infrastructure also squeezes out environmental protection funding [65], making it difficult to carry out environmental restoration work. UGLUE is reduced. At present, infrastructure investment is still one of the important means for local governments to promote economic growth. From January to May 2024, the national fixed asset investment is 188006 billion yuan. Among them, infrastructure investment increases by 5.7% year-on-year, 1.7 percentage points higher than the total investment. In the future, the government should optimize the investment structure and gradually reduce its reliance on traditional infrastructure investment. Existing studies have mainly focused on the impact of economic growth targets on environmental pollution [66], but not on their impact on UGLUE. Currently, there is limited understanding of how economic growth policies affect urban land use patterns in practice. This study fills this gap.

The estimated coefficient of soft constraints is positive at the 5% significance level, indicating that soft constraints improve UGLUE. Traditional studies usually analyzed the effects of economic growth target constraints as a whole [67]. They ignored the potential environmental benefits of soft policy constraints. Wang and Lei [68] emphasized that economic policies need to balance growth and environmental protection. However, few studies have linked soft constraints to UGLUE. The soft constraint characteristics of economic growth targets enable local governments to pay more attention to long-term interests when formulating economic development plans. When planning urban land use, the government gives priority to environmental protection factors, such as setting up ecological protection areas and promoting green buildings, so as to improve UGLUE. Under the soft constraint of economic growth targets, Chengdu implemented the “Urban Green Heart Plan” in 2021 to increase the ecological benefits of land. In addition, Chengdu has continuously improved the city’s green coverage rate by implementing a series of ecological projects, such as the Ring City Ecological Zone and Jinjiang Park. By 2024, the green coverage rate of Chengdu’s built-up area has increased to 44.7%.

Regarding the control variables, the estimated coefficients of economic development level and population size are significantly positive. With economic growth, governments and enterprises invest more funds in the research and development of advanced land use technologies, thus promoting the intensive use of urban land. People are paying more and more attention to their own health and environmental quality. They not only adopt green lifestyles themselves, but also encourage enterprises to produce technology-intensive rather than resource-intensive products and reduce pollutant emissions. The expansion of population size can bring about “scale effect” and “agglomeration effect”, which helps share knowledge and technology, thereby improving UGLUE. The degree of openness and labor distortion have a negative impact on UGLUE. At present, China mainly relies on highly polluting resource-intensive industries to drive exports, which consumes a large amount of land resources and increase pollutant emissions. The concentration of labor in pollution-intensive industries leads to a shortage of labor in technology-intensive industries.

### 5.3. Robustness test

#### 5.3.1. Shorten research period.

To avoid the impact of the epidemic from interfering with research conclusions, the sample data in 2020 and 2021 are eliminated and then regression analysis is performed (Table 4). The results demonstrate the robustness of benchmark regression results.

**Table 4:** Robustness test.

	Shorten research period			Excluding municipalities			Adding year fixed effects		
	(1)	(2)	(3)	(4)	(5)	(6)	(7)	(8)	(9)
EG	‐0.0170***			‐0.0350***			‐0.0105**		
	(0.0026)			(0.0023)			(0.0048)		
EHC		‐0.0146*			‐0.0281			‐0.0478**	
		(0.0082)			(0.0173)			(0.0193)	
ESC			0.0180*			0.0338**			‐0.0506***
			(0.0106)			(0.0152)			(0.0176)
lnGDPP	0.221***	0.0926*	0.0982*	0.382***	0.167***	0.173***	0.251***	0.278***	0.276***
	(0.0591)	(0.0560)	(0.0564)	(0.0492)	(0.0488)	(0.0489)	(0.0557)	(0.0537)	(0.0537)
OPEN	‐0.201**	‐0.291***	‐0.284***	0.148*	‐0.0336	‐0.0229	‐0.0766	‐0.0781	‐0.0798
	(0.0868)	(0.0863)	(0.0866)	(0.0812)	(0.0833)	(0.0834)	(0.0875)	(0.0875)	(0.0874)
LD	0.0052	0.0116	0.0114	‐0.0168**	‐0.0068	‐0.0072	‐0.0008	‐0.0006	‐0.0009
	(0.0083)	(0.0084)	(0.0084)	(0.0079)	(0.0082)	(0.0082)	(0.0089)	(0.0089)	(0.0089)
lnPOP	0.494***	0.494***	0.489***	0.753***	0.758***	0.757***	0.401***	0.452***	0.446***
	(0.146)	(0.148)	(0.148)	(0.123)	(0.128)	(0.127)	(0.141)	(0.139)	(0.139)
Constant	‐3.903***	‐2.753**	‐2.797***	‐6.859***	‐5.010***	‐5.103***	‐3.981***	‐4.469***	‐4.360***
	(1.079)	(1.073)	(1.073)	(0.917)	(0.943)	(0.943)	(1.052)	(1.026)	(1.026)
R^2^	0.03	0.014	0.015	0.085	0.017	0.017	0.246	0.247	0.247
N	2730	2730	2730	3228	3228	3228	3276	3276	3276

Standard errors in parentheses

***p<0.01.

**p<0.05.

*p<0.1.

#### 5.3.2. Excluding municipalities.

There are obvious differences between the administrative levels of municipalities and general prefecture-level cities. We delete Beijing, Tianjin, Shanghai and Chongqing to reduce the estimation bias caused by the large systematic differences between samples.

#### 5.3.3. Replacing model.

In order to eliminate the estimation bias caused by omitted factors, this paper further controls the year fixed effect on the basis of baseline regression. The estimated results are consistent with the baseline regression results. This shows that the baseline regression results have a certain degree of robustness.

### 5.4. Endogeneity test

UGLUE may be affected by non-quantifiable indicators such as cultural concepts and institutional environment. Ignoring non-quantifiable indicators may cause endogenous problems. In addition, the government may set corresponding economic growth targets based on its forecasts of local land use, leading to reverse causality problems. To solve the endogeneity problem caused by omitted variables and reverse causality, this paper uses instrumental variable method to test. According to the instrumental variable selection criteria, this paper uses the economic growth target of the province where each city is located as instrumental variable. In terms of relevance, China has a “promotion tournament” with GDP as the assessment standard [69]. To win the “promotion tournament”, local governments “add layers of weight” to the economic growth targets set by higher-level governments, which is consistent with the relevance of instrumental variables. In terms of exogeneity, the UGLUE in individual prefecture-level cities has little correlation with the mean economic growth target of the province where they are located. For the hard and soft constraints of economic growth targets, this paper uses the interaction term of the number of prefecture-level cities in the province where the prefecture-level cities are located (related to individual changes) and the average of the national economic growth targets for next two years (related to time) as its instrumental variables.

As shown in Table 5, the regression coefficients of instrumental variables all pass the 1% significance test, indicating that they have a certain correlation. The regression coefficient of economic growth target value and its hard constraints is still significantly positive. The regression coefficient of soft constraints is still significantly negative. This demonstrates the robustness of estimation results. The test results show that the instrumental variables have certain rationality and effectiveness.

**Table 5:** Endogeneity test.

	(1)	(2)	(3)	(4)	(5)	(6)
EG		-0.0458***				
		(0.0030)				
EHC				-1.240***		
				(0.116)		
ESC						0.784***
						(0.0651)
lnGDPP	0.6195***	0.409***	0.0075	0.390***	-0.0275	0.402***
	(0.2083)	(0.0685)	(0.0522)	(0.0929)	(0.0567)	(0.0806)
OPEN	-0.8089**	0.218***	-0.1104	0.119	-0.0530	0.297***
	(0.4041)	(0.0797)	(0.0925)	(0.135)	(0.1048)	(0.111)
LD	-0.0402	-0.0265**	-0.0073	-0.0337*	0.0108	-0.0331*
	(0.0304)	(0.0118)	(0.0084)	(0.0185)	(0.0124)	(0.0180)
lnPOP	0.9008**	0.970***	-0.2021*	0.678***	0.1540	0.808***
	(0.4525)	(0.182)	(0.1105)	(0.230)	(0.1482)	(0.203)
PEG	0.9590***					
	(0.0116)					
Quantity×GEG×WCEG			0.0354***		-0.0561***	
			(0.0024)		(0.0027)	
R^2^	0.8539	0.0678	0.6710	0.0901	0.579	0.0721
N	3228	3228	3228	3228	3228	3228
F test	1548.61***		215.33***		417.74***	
Kleibergen-Paap rk LM statistic		931.45***		194.797***		353.091***
Cragg-Donald Wald F statistic		6826.753		215.325		417.743
Stock-Yogo bias critical value		16.38(10%)		16.38(10%)		16.38(10%)

Standard errors in parentheses.

***p<0.01.

**p<0.05.

*p<0.1.

### 5.5. Heterogeneity analysis

#### 5.5.1. Heterogeneity analysis based on geographical location.

Due to China’s vast territory, the constraints of local EGTM are also different, which may have different impacts on UGLUE. According to the standards given by the National Bureau of Statistics, this article divides 273 prefecture-level cities into three regions: Eastern, central and western. Table 6 shows that the economic growth target value has the greatest negative impact on UGLUE in central regions, followed by eastern regions and has the least negative impact on UGLUE in western regions. Compared with western region, the central region has more developed industries and manufacturing industries, which tend to be more dependent on land resources. Under the pressure of economic growth targets, local governments are more inclined to support the development of these industries. Land resources are over-exploited and inefficiently used.

**Table 6:** Heterogeneity analysis based on geographical location.

	Eastern regions			Central regions			Western regions		
	(1)	(2)	(3)	(4)	(5)	(6)	(7)	(8)	(9)
EG	-0.0329***			-0.0492***			-0.0229***		
	(0.0065)			(0.0042)			(0.0039)		
EHC		-0.0459			-0.0211			0.0250	
		(0.0406)			(0.0318)			(0.0313)	
ESC			0.0249*			0.0763***			-0.0322
			(0.0148)			(0.0277)			(0.0289)
lnGDPP	-0.0963	-0.266**	-0.275**	0.845***	0.484***	0.513***	0.451***	0.320***	0.314***
	(0.119)	(0.115)	(0.115)	(0.0907)	(0.0905)	(0.0907)	(0.0899)	(0.0894)	(0.0897)
OPEN	0.0601	0.0204	0.0190	0.959***	0.602**	0.643***	0.229	0.0465	0.0242
	(0.154)	(0.155)	(0.156)	(0.232)	(0.244)	(0.244)	(0.197)	(0.199)	(0.199)
LD	-0.0259**	-0.0255*	-0.0251*	0.0461	0.135***	0.122***	-0.0196	0.0041	0.0058
	(0.0129)	(0.0131)	(0.0131)	(0.0301)	(0.0310)	(0.0312)	(0.0253)	(0.0255)	(0.0256)
lnPOP	2.135***	2.488***	2.529***	0.392**	0.230	0.239	0.364	0.315	0.317
	(0.391)	(0.389)	(0.389)	(0.182)	(0.192)	(0.192)	(0.272)	(0.278)	(0.277)
Constant	-10.51***	-11.14***	-11.33***	-9.196***	-5.114***	-5.491***	-5.323***	-3.998**	-3.916**
	(2.848)	(2.879)	(2.878)	(1.491)	(1.539)	(1.540)	(1.732)	(1.752)	(1.753)
R^2^	0.091	0.072	0.071	0.152	0.048	0.055	0.058	0.020	0.021
N	1200	1200	1200	1188	1188	1188	888	888	888

Standard errors in parentheses.

***p<0.01.

**p<0.05.

*p<0.1.

#### 5.5.2. Heterogeneity analysis based on resource endowment.

According to the National Sustainable Development Plan for Resource-Based Cities (2013–2020) issued by the State Council, this paper divides China’s 273 cities into 108 resource-based cities and 165 non-resource-based cities, and further analyzes the heterogeneous impact of EGTM on the UGLUE in cities with different resource endowments. Table 7 shows that the negative impact of economic growth target value on the UGLUE in resource-based cities is greater than that in non-resource-based cities. Resource-based cities take the mining and processing of the region’s unique natural resource endowments (such as minerals, forests and other natural resources) as leading industries. Under the pressure of economic growth targets, compared with non-resource-based cities, resource-based city governments are more inclined to achieve economic growth by supporting industries such as resource mining and processing, thereby exacerbating environmental pollution and reducing UGLUE. Soft constraints can significantly improve the UGLUE of non-resource-based cities, but the impact on UGLUE of non-resource-based cities is not significant. The industrial structure of non-resource-based cities is diversified, covering multiple fields such as manufacturing, services, and high-tech industries. Under the soft constraints of economic growth targets, non-resource-based cities can adjust their industrial structure more flexibly and promote the development of emerging industries, thereby improving UGLUE. Resource-based cities have a single industrial structure. Even if soft constraints are set on economic growth targets, local governments cannot effectively improve UGLUE.

**Table 7:** Heterogeneity analysis based on resource endowment.

	Resource-based cities			Non-resource cities		
	(1)	(2)	(3)	(4)	(5)	(6)
EG	-0.0236***			-0.0181***		
	(0.0037)			(0.0041)		
EHC		-0.0104			-0.0380	
		(0.0282)			(0.0289)	
ESC			-0.0282			0.0628**
			(0.0249)			(0.0253)
lnGDPP	0.348***	0.128*	0.111	0.288***	0.148	0.155*
	(0.0756)	(0.0683)	(0.0691)	(0.0907)	(0.0933)	(0.0931)
OPEN	0.381**	0.174	0.159	0.0903	-0.0859	-0.0748
	(0.169)	(0.168)	(0.169)	(0.123)	(0.126)	(0.126)
LD	-0.0163*	-0.0109	-0.0102	-0.0277	-0.0153	-0.0161
	(0.0098)	(0.0099)	(0.0099)	(0.0187)	(0.0193)	(0.0193)
lnPOP	-0.182	-0.230	-0.232	1.847***	2.037***	2.018***
	(0.180)	(0.183)	(0.183)	(0.236)	(0.245)	(0.244)
Constant	-1.202	1.070	1.249	-12.51***	-12.62***	-12.64***
	(1.317)	(1.289)	(1.295)	(1.789)	(1.856)	(1.853)
R^2^	0.039	0.021	0.07	0.110	0.049	0.052
N	1296	1296	1296	1980	1980	1980

Standard errors in parentheses.

***p<0.01.

**p<0.05.

*p<0.1.

According to Cleary [70], this paper employs the Fisher’s combined probability test method for testing differences between groups of coefficients (Table 8). The number of sampling iterations is 1,000. The p-values for the tests on differences between groups for economic growth target values and their soft constraints are less than 0.1, indicating that the impact of economic growth target values and their soft constraints on UGLUE varies significantly across different geographical locations and cities with different resource endowments. The hard constraints cannot pass the inter-group difference test, suggesting that the impact of hard constraints on UGLUE does not differ significantly across different geographical locations and resource-endowed cities.

**Table 8:** Test for differences in coefficients between groups.

	EG	EHC	ESC
Eastern regions versus Central regions	0.020***	0.012	0.035**
Eastern regions versus Western regions	0.022***	0.022	0.026**
Central regions versus Western regions	0.031**	0.034*	0.011***
Resource-based cities versus Non-resource-based cities	0.025***	0.026	0.095*

### 5.6. Mechanism test

#### 5.6.1. Green technology innovation.

To test the impact of EGTM on UGLUE through green technology innovation, this study selects the number of green patent applications as an indicator of green technology innovation [71]. Group regression analysis is conducted using this indicator. To avoid any influence of EGTM on green technology innovation within the sample period that might bias the grouping results, the study divides the sample into two groups. The groups are categorized as high and low based on the median level of green technology innovation at the beginning of the sample period (2010) for regression analysis.

As shown in Table 9, regions with higher levels of green technology innovation experience a greater impact of EGTM on UGLUE compared to regions with lower levels of green technology innovation. EGTM can influence UGLUE through green technology innovation, which is similar to the conclusion of Guo et al. [72]. The difference is that their study focused on environmental pollution, while this study takes into account both land use and environmental pollution. This study provides a more comprehensive perspective on the relationship between economic policies, technological innovation, and sustainable development.

**Table 9:** Mechanism test (green technology innovation).

	High level of green technology innovation			Low level of green technology innovation		
	(1)	(2)	(3)	(4)	(5)	(6)
EG	-0.0650***			-0.0208***		
	(0.0048)			(0.0035)		
EHC		-0.0593**			-0.0018	
		(0.0279)			(0.0317)	
ESC			0.0728***			-0.0141
			(0.0250)			(0.0270)
lnGDPP	0.252***	0.131**	0.131**	0.209***	0.121**	0.117**
	(0.0583)	(0.0593)	(0.0592)	(0.0607)	(0.0592)	(0.0595)
OPEN	-0.111	-0.226**	-0.219**	-0.168*	-0.234**	-0.236**
	(0.0946)	(0.0969)	(0.0969)	(0.0970)	(0.0969)	(0.0970)
LD	-0.0218**	-0.0134	-0.0137	-0.0158	-0.0127	-0.0125
	(0.0097)	(0.0100)	(0.0100)	(0.0099)	(0.0100)	(0.0100)
lnPOP	0.593***	0.947***	0.937***	1.074***	0.966***	0.963***
	(0.154)	(0.156)	(0.156)	(0.156)	(0.156)	(0.156)
Constant	-4.599***	-5.720***	-5.704***	-7.186***	-5.747***	-5.699***
	(1.125)	(1.155)	(1.154)	(1.174)	(1.156)	(1.159)
R^2^	0.074	0.020	0.022	0.030	0.019	0.019
N	1632	1632	1632	1644	1644	1644

Standard errors in parentheses.

***p<0.01.

**p<0.05.

*p<0.1.

To achieve economic growth, governments often increase investment in infrastructure, which reduces the availability of funds in the market. Consequently, financial institutions raise loan interest rates. This increases financing costs for companies [73] and makes it challenging to sustain investment in green technology development. In addition, local governments vigorously support the real estate industry to promote economic growth, leading to rising housing prices. In pursuit of short-term profits, some companies invest funds originally intended for green technology research and development into the real estate market, further squeezing out innovation funds.

The soft constraint of economic growth targets prompts local governments to take environmental protection into account when investing in infrastructure, and encourages companies to increase investment in green technology research. Green technology innovation can reduce the consumption of fossil energy from production source and improve the degree of pollutant purification in end-of-pipe treatment, thereby improving UGLUE [74].

#### 5.6.2. Upgrading of industrial structure.

To test how EGTM influences UGLUE through the upgrading of industrial structures, this paper selects the ratio of the tertiary industry value-added to the secondary industry value-added as the measure of industrial upgrading [75]. This indicator is used for group regression analysis. The median level of industrial upgrading at the start of the sample period (2010) is used to divide the sample into two groups: high and low.

As shown in Table 10, EGTM has a greater impact on UGLUE in regions with a high level of industrial upgrading than in regions with a low level of industrial upgrading. EGTM can affect UGLUE through the upgrading of industrial structure. Ren et al. [34] pointed out that there is an interactive relationship between EGTM and industrial structure upgrading. However, they did not explore the relationship between EGTM, industrial structure upgrading and land use in depth. In pursuit of short-term economic growth, local governments excessively tilt production factors such as capital and land toward heavy industry enterprises that lack innovation momentum [76]. Emerging industries that require large initial investments and long growth cycles are restricted from development. The pace of industrial structure upgrading slows down.

**Table 10:** Mechanism test (upgrading of industrial structure).

	High level of industrial upgrading			Low level of industrial upgrading		
	(1)	(2)	(3)	(4)	(5)	(6)
EG	-0.0503***			-0.0229***		
	(0.0041)			(0.0039)		
EHC		-0.0588**			-0.0100	
		(0.0297)			(0.0295)	
ESC			0.0239***			0.0418
			(0.0058)			(0.0262)
lnGDPP	0.177***	0.123**	0.121**	0.239***	0.122**	0.134**
	(0.0580)	(0.0592)	(0.0592)	(0.0622)	(0.0594)	(0.0598)
OPEN	-0.0596	-0.228**	-0.230**	-0.197**	-0.234**	-0.226**
	(0.0957)	(0.0969)	(0.0970)	(0.0966)	(0.0969)	(0.0970)
LD	-0.0190*	-0.0130	-0.0129	-0.0164	-0.0129	-0.0135
	(0.0097)	(0.0100)	(0.0100)	(0.0099)	(0.0100)	(0.0100)
lnPOP	0.894***	0.952***	0.961***	0.986***	0.965***	0.963***
	(0.152)	(0.156)	(0.156)	(0.155)	(0.156)	(0.156)
Constant	-5.676***	-5.673***	-5.734***	-6.959***	-5.759***	-5.879***
	(1.128)	(1.156)	(1.156)	(1.167)	(1.156)	(1.158)
R^2^	0.064	0.020	0.019	0.03	0.019	0.02
N	1632	1632	1632	1644	1644	1644

Standard errors in parentheses.

***p<0.01.

**p<0.05.

*p<0.1.

When the setting of economic growth targets shifts from “hard constraints” to “soft constraints”, Local governments have more flexibility in resource allocation. This enables them to rationally allocate resources while promoting economic growth. Specifically, they can invest financial resources in technology research and development and environmental protection industries. The upgrading of industrial structure is promoted, which can guide low-pollution industries to gather in the city center and traditional high-pollution industries to move to the periphery of city [77]. The economic development of urban land and environmental protection are balanced.

### 5.7. Threshold effect analysis

#### 5.7.1. Threshold effect test.

This paper adopts the Bootstrap self-sampling method and repeats the sampling 300 times. The test results corresponding to each threshold quantity are shown in Table 11. The single threshold tests of explanatory variables (EG, EHC and ESC) all pass 10% significance test, but the double threshold tests fail to pass significance test. This shows that environmental regulation has a single threshold effect in the impact of EGTM on UGLUE.

**Table 11:** Threshold effect test results.

	Threshold number	Threshold value	Fstat	Prob	10%	5%	1%
EG	Single	0.3268	31.9	0.0767	30.1942	34.0416	47.6272
	Double		3.73	0.7033	10.1158	12.1736	15.8252
EHC	Single	0.3268	16.73	0.0533	14.5316	16.7892	22.5105
	Double		6.67	0.3033	10.1354	11.4322	16.642
ESC	Single	0.3268	11.96	0.0667	10.636	12.7705	17.9401
	Double		7.32	0.3533	11.1005	13.5204	17.6752

**Fig 4:**
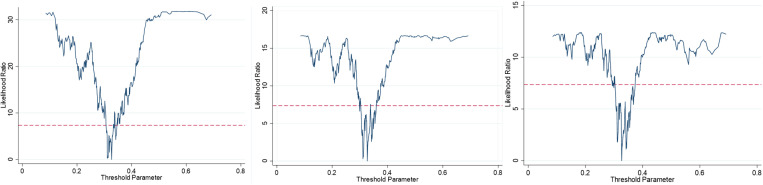
LR graph of threshold value.

As can be seen from Fig 4, when environmental regulation is used as the threshold variable, the threshold values of economic growth management (EG, EHC and ESC) for UGLUE are all 0.3268.

#### 5.7.2. Panel threshold model estimation results.

Table 12 shows the estimation results of panel threshold model. When the intensity of environmental regulation is lower than 0.3268. The estimated coefficients of economic growth target value and its hard constraints are negative at the 1% significance level. The estimated coefficient of soft constraints is positive but not significant. When the intensity of environmental regulation is higher than 0.3268, the absolute value of economic growth target regression coefficient decreases. The estimated coefficient of hard constraints becomes positive. The estimated coefficient of soft constraints has increased and passed the 5% significance test. When the intensity of environmental regulation is low, local governments tend to pay more attention to economic growth, resulting in a large amount of land resources being used for high-pollution, high-value industries. As the intensity of environmental regulation increases, the governments guide enterprises to develop in the direction of low pollution and high value by setting up environmental barriers and providing preferential policies.

**Table 12:** Panel threshold model regression results.

	(1)	(2)	(3)
EG(ER≤0.3268)	-0.0405***		
	(0.0045)		
EG(ER>0.3268)	-0.0324***		
	(0.0042)		
EHC(ER≤0.3268)		-0.0605***	
		(0.0208)	
EHC(ER>0.3268)		0.0432	
		(0.0266)	
ESC(ER≤0.3268)			0.0311
			(0.0205)
ESC(ER>0.3268)			0.0427**
			(0.0210)
lnGDPP	0.376***	0.158**	-0.0364
	(0.0808)	(0.0775)	(0.0714)
OPEN	-0.0538	-0.241	-0.205
	(0.165)	(0.173)	(0.176)
LD	-0.0152	-0.0055	0.0018
	(0.0124)	(0.0128)	(0.0130)
lnPOP	0.688**	0.710**	0.417
	(0.282)	(0.306)	(0.279)
Constant	-6.389***	-4.609**	-0.833
	(1.812)	(1.901)	(1.694)
R^2^	0.093	0.028	0.130
N	3276	3276	3276

Standard errors in parentheses.

***p<0.01.

**p<0.05.

*p<0.1.

### 5.8. Spatial spillover effect analysis

Existing research mostly focuses on the direct effects of EGTM [35]. Few studies have explored how economic growth targets influence neighboring regions’ UGLUE through spatial interactions. In fact, the economic growth targets set by various levels of government in China are not independent but form an EGTM system. There are complex spatial strategic interactions among these targets. Therefore, it is necessary to introduce spatial econometric models to analyze the impact of spatial strategic interactions between governments at the same level on UGLUE.

#### 5.8.1. Spatial autocorrelation test.

Table 13 shows the global Moran’s I values of UGLUE, EG, EHC and ESC from 2010 to 2021. From 2010 to 2021, the global Moran’s I value of UGLUE, economic growth target value, hard constraints and soft constraints is significantly positive, which shows that UGLUE, economic growth target value, hard constraints and soft constraints all have positive spatial correlation.

**Table 13:** Global Moran’s I.

	UGLUE	EG	EHC	ESC
2010	-0.005	0.083***	0.089***	0.068***
2011	-0.004	0.102***	0.017**	0.007
2012	-0.006	0.095***	-0.005	-0.001
2013	0.017**	0.077***	0.008	0.019**
2014	0.013*	0.108***	0.007	0.021***
2015	-0.002	0.162***	0.014**	-0.007
2016	0.011*	0.150***	0.017**	-0.005
2017	0.011*	0.123***	0.005	0.023***
2018	0.013*	0.134***	0.009	0.011*
2019	0.021***	0.145***	0.013*	0.014**
2020	0.027***	0.087***	0.018**	0.010
2021	0.023***	0.095***	0.019**	-0.012

***p<0.01.

**p<0.05.

*p<0.1.

#### 5.8.2. SDM estimation results.

According to the test results in Table 14, this paper chooses the estimation results of SDM for interpretation. The spatial lag coefficient of economic growth target value and its hard constraints is negative at the 1% significance level, indicating that the economic growth target and its hard constraints have a negative spatial spillover effect on UGLUE. There is a “race to the top” among local governments in setting economic growth targets, which increases the pressure on local economic growth [78]. Governments in different regions often set higher growth targets to avoid falling behind other regions in economic growth. This competitive target setting increases the pressure on local economic growth. Under tremendous pressure, local governments focus on economic growth, ignore environmental protection [79]. UGLUE is reduced. In the future, environmental protection should be included in government performance evaluation system, so that there will be not only economic competition but also environmental competition among governments. Based on this, local governments have to consider both the economic and ecological benefits of land use.

**Table 14:** Spatial econometric model estimation results.

	(1)SAR	(2)SEM	(3)SDM	(4)SAR	(5)SEM	(6)SDM	(7)SAR	(8)SEM	(9)SDM
EG	-0.0007	-0.0014	-0.0106***						
	(0.0021)	(0.0038)	(0.0039)						
EHC				-0.0405***	-0.0503***	-0.0491***			
				(0.0139)	(0.0143)	(0.0144)			
ESC							0.0352***	0.0441***	0.0471***
							(0.0122)	(0.0131)	(0.0131)
lnGDPP	0.284***	0.385***	0.361***	0.272***	0.348***	0.365***	0.268***	0.346***	0.368***
	(0.0409)	(0.0472)	(0.0485)	(0.0391)	(0.0443)	(0.0474)	(0.0392)	(0.0443)	(0.0475)
OPEN	-0.112*	-0.103	-0.107	-0.120*	-0.107	-0.146**	-0.127**	-0.109	-0.139**
	(0.0646)	(0.0685)	(0.0689)	(0.0638)	(0.0672)	(0.0686)	(0.0639)	(0.0672)	(0.0686)
LD	0.0016	0.0031	0.0037	0.0027	0.0038	0.0052	0.0029	0.0034	0.0052
	(0.0066)	(0.0067)	(0.0067)	(0.0065)	(0.0067)	(0.0067)	(0.0066)	(0.0067)	(0.0067)
lnPOP	0.291***	0.453***	0.365***	0.297***	0.331***	0.434***	0.295***	0.331***	0.408***
	(0.103)	(0.112)	(0.113)	(0.103)	(0.109)	(0.112)	(0.103)	(0.109)	(0.112)
W*EG			-0.0200***						
			(0.0068)			-0.0551**			
W*EHC						(0.0211)			
W*ESC									0.125**
									(0.0566)
W*lnGDPP			-0.581***			-0.369**			-0.512***
			(0.198)			(0.179)			(0.189)
W*OPEN			0.856*			-0.0738			-0.491
			(0.460)			(0.440)			(0.464)
W*LD			-0.0466			0.162***			0.199***
			(0.0427)			(0.0409)			(0.0426)
W*lnPOP			0.0888			-3.331***			-3.194***
			(0.577)			(0.529)			(0.535)
R^2^	0.066	0.0145	0.126	0.065	0.069	0.156	0.065	0.069	0.1894
N	3276	3276	3276	3276	3276	3276	3276	3276	3276
Wald test spatial lag			29.75***			65.82***			71.45***
Wald test spatial error			13.53**			66.60***			73.80***
LR test spatial lag			30.17***			14.48***			7.42***
LR test spatial error			119.34***			29.56***			21.80***

Standard errors in parentheses.

***p<0.01.

**p<0.05.

*p<0.1.

## 6. Conclusion and policy recommendations

Based on sample data from 273 cities in China from 2010 to 2021, this paper uses panel data model to empirically test the impact of EGTM (including economic growth target value, its hard constraints and soft constraints) on UGLUE and its transmission mechanism. The panel threshold model is used to verify the threshold role of environmental regulation in the relationship between EGTM and UGLUE. The SDM is used to verify the spatial spillover effect of EGTM on UGLUE. The conclusions are as follows: (1) The local economic growth target value and its hard constraints have a negative impact on UGLUE. The soft constraint of “leaving room for maneuver” is conducive to improving UGLUE. (2) Green technology innovation and industrial structure upgrading are the transmission mechanisms of EGTM affecting UGLUE. (3) The intensity of environmental regulation has a threshold effect on the impact of EGTM on UGLUE. As the intensity of environmental regulation increases, the negative impact of economic growth target value and its hard constraints on UGLUE weakens. The positive impact of economic growth target soft constraints on UGLUE increases. (4) EGTM has a spatial spillover effect on UGLUE. The economic growth target value and its hard constraints of surrounding areas can reduce the UGLUE in local region, while its soft constraints can improve the UGLUE in local region. (5) Regarding geographical location heterogeneity, economic growth targets have the greatest negative impact on UGLUE in the central region, followed by the eastern region, and have the least negative impact on UGLUE in the western region. Regarding resource endowment heterogeneity, the negative impact of economic growth targets on UGLUE in resource-based cities is greater than that in non-resource-based cities. The positive impact of soft constraints on UGLUE in resource-based cities is smaller than that in non-resource-based cities.

Based on the research conclusions, this paper puts forward the following policy recommendations:

(1) Reform the official assessment system and downplay the importance of GDP growth in official performance assessment. At present, some local governments are still deeply trapped in the concept of “GDP only”, which has led to the distortion of government fiscal investment and the mismatch of land resources, seriously threatening the green use of urban land. To solve this problem, we should actively introduce assessment indicators such as environmental quality and land use. Based on this, local governments have to consider ecological protection while developing the economy. In addition, the soft constraint method of “leaving room” is more often adopted in setting economic growth targets, which makes local governments pay more attention to the ecological benefits of land in policy making.
(2) Strengthen environmental regulation. The government should strictly control the entry of high-polluting enterprises into the market and fundamentally abandon high-polluting and high-energy-consuming production methods. Meanwhile, the use of clean energy is encouraged and green subsidies are increased to incentivize corporate transformation. These measures are conducive to fully releasing the weakening effect of environmental regulation in the process of economic growth goals negatively affecting UGLUE.
(3) Establish a differentiated government performance evaluation system. There is a phenomenon of “competing for the top” among regions when setting economic growth targets, which is not conducive to improving UGLUE. Therefore, higher-level governments should abandon the traditional “one-size-fits-all” approach to government performance evaluation. Based on the actual environmental conditions of each jurisdiction, the effectiveness of environmental governance should be selectively incorporated into the performance evaluation system. Based on this, local governments can be guided to form a healthy competition and jointly promote the green use of urban land.

The limitations of this study are primarily reflected in two aspects. First, the analysis primarily relies on quantifiable data to examine the relationship between economic policy and UGLUE, failing to fully capture the cultural, political, and social factors that affect UGLUE. Non-quantifiable factors such as local officials’ policy preferences and regional cultural differences could significantly impact UGLUE. Second, the data used in this study are limited to the period from 2010 to 2021. The data within this timeframe may not fully reflect the long-term effects and time lags following economic policy adjustments. Future research could incorporate qualitative research methods, such as case studies, expert interviews, or focus group discussions, to delve deeper into the impact of non-quantifiable factors on UGLUE. Additionally, extending the timeframe could help observe the long-term effects of policy changes on UGLUE.

## Supporting information

S1 Data Data. (XLSX)
